# Genetic predisposition to fracture non-union: a case control study of a preliminary single nucleotide polymorphisms analysis of the BMP pathway

**DOI:** 10.1186/1471-2474-12-44

**Published:** 2011-02-10

**Authors:** Rozalia Dimitriou, Ian M Carr, Robert M West, Alexander F Markham, Peter V Giannoudis

**Affiliations:** 1 Academic Unit of Trauma and Orthopaedic Surgery, LIMM Section Musculoskeletal Disease, Clarendon Wing, Leeds Teaching Hospitals NHS Trust, Great George Street, Leeds LS1 3EX, UK; 2 Section of Translational Medicine, Leeds Institute of Molecular Medicine, University of Leeds, Leeds, UK; 3 Biostatistics Unit, Division of Epidemiology and Biostatistics, Leeds Institute of Genetics, University of Leeds, Leeds, UK

## Abstract

**Background:**

Despite the known multi-factorial nature of atrophic fracture non-unions, a possible genetic predisposition for the development of this complication after long bone fractures remains unknown. This pilot study aimed to address this issue by performing a preliminary SNP analysis of specific genes known to regulate fracture healing.

**Methods:**

A total of fifteen SNPs within four genes of the Bone Morphogenetic Protein (BMP) pathway (*BMP-2, BMP-7*, *NOGGIN *and *SMAD6*) were examined, in 109 randomly selected patients with long bone fractures as a result of motor vehicle accident, fall or direct blow. There were sixty-two patients with atrophic non-union and forty-seven patients (54 fractures) with uneventful fracture union. Overall SNPs frequencies were computed with respect to patient's age, gender, smoking habits, fracture-associated parameters and the use of nonsteroidal anti-inflammatory drugs (NSAIDs), and tested for their association to the impaired bone healing process, using binary logistic regression (STATA 11.1; StataCorp, Texas USA).

**Results:**

Statistical analysis revealed age to be an important covariate in the development of atrophic non-union (*p *= 0.01, OR 1.05 [per year]), and two specific genotypes (G/G genotype of the rs1372857 SNP, located on *NOGGIN *and T/T genotype of the rs2053423 SNP, located on *SMAD6*) to be associated with a greater risk of fracture non-union (*p *= 0.02, OR 4.56 and *p *= 0.04, OR 10.27, respectively, after adjustment for age).

**Conclusions:**

This is the first clinical study to investigate the potential existence of genetic susceptibility to fracture non-union. Even though no concrete conclusions can be obtained from this pilot study, our results indicate the existence of a potential genetically predetermined impairment within the BMP signalling cascade, initiated after a fracture and when combined with other risk factors could synergistically increase the susceptibility of a patient to develop non-union. Further research is desirable in order to clarify the genetic component and its role and interaction with other risk factors in the development of atrophic long bone non-union, as simple genetic testing may contribute to the early identification of patients at risk in the future and the on-time intervention at the biologic aspects of bone healing.

## Background

Although bone possesses great intrinsic potential for regeneration and repair, impaired healing response (delayed union/non-union) following a fracture has been reported to range between 5-10% [1]. Several factors have been associated with non-union of fractures including poor mechanical stability, the presence of a gap at the fracture site, extensive soft tissue damage and open fractures, administration of pharmacological agents, such as NSAIDs, and smoking [2,3]. However, the possible role of genetic variations on the fracture healing response among individuals and a potential genetic predisposition of atrophic non-union of fractures remain unknown. Recently, with the completion of the human genome project, the importance of genes as causes of diseases or as predisposing factors has become indisputable [4-8]. The observed polymorphisms demonstrated for a specific disease process are of different nature. Some of them are mutations located within endonuclease restriction sites, others are single nucleotide polymorphisms (SNPs: DNA variations at a single nucleotide) or consist of insertions or deletions of larger fragments as detected by polymerase chain reaction technique (PCR) [9].

During fracture healing and bone repair, a number of molecules present on the extracellular matrix regulate the cascade of events at the molecular and cellular level. Among other molecules, the group of bone morphogenetic proteins (BMPs), which are members of the transforming growth factor-beta (TGF-β) superfamily, are being extensively studied, as they exhibit powerful osteoinductive properties by inducing both proliferation and differentiation of mesenchymal stem cells (MSCs) and osteoprogenitor cells [10,11]. Currently, a number of different human BMPs have been identified, according to their primary amino acid sequence. BMP-2 and BMP-7 are two widely studied members and are already in clinical use as osteoinductive molecules. BMP signal transduction is induced via serine/threonine kinase receptors, initiating the intracellular Smad signalling pathway. The Smad family includes three groups: the signal-transducing receptor regulated (R-Smads: 1, 2, 3, 5, 8), the common mediator (co-Smad or Smad4), and the inhibitory ones (I-Smads: 6 and 7) [12,13]. Recently, a number of molecules displaying inhibitory properties and regulating the BMP pathway, as well as other pathways during bone regeneration, have been also identified [14]. A well-known extracellular inhibitor of BMPs is noggin which antagonises their actions by preventing their binding with the BMP receptors [15].

The purpose of this pilot study was to investigate whether genetic variants within genes of the fracture healing cascade, can be correlated with an impaired fracture healing response, by performing a preliminary SNP analysis of the BMP pathway. The primary hypothesis was that specific SNPs may be associated with the development of atrophic fracture non-unions. Other parameters known to predispose to non-union were also evaluated.

## Methods

After approval by the Local Research Ethics Committee of Leeds, (East) Research Ethics Committee (Project No: 03/220), we retrospectively studied 109 patients with long bone fractures admitted and treated in the author's institution from 2005 to 2007. The patients were selected randomly from the hospital database and those who met the inclusion criteria of the study were invited to participate. Only British born Caucasians were included, in an effort to have a genetically homogenous cohort of patients. All patients had initially sustained a long bone fracture as a result of a road traffic accident, a fall from height or a direct blow. All fractures were long bone fractures (open or closed, and diaphyseal or diaphyseal+metaphyseal fractures).

Non-union was defined as the cessation of all healing processes and failure to achieve union after the expected period of time, as seen clinically and radiologically. Union was defined as painless, without movement fracture site or painless full weight bearing in case of fractures of the lower extremity; with the presence of bridging callus in three out of four cortices in two radiological planes [2]. During the selection of union patients, a quota sampling regarding open vs closed fractures (approximately 50% of each) has been performed in an effort to match the high incidence of open fractures seen in non-union patients. Exclusion criteria included children and patients with a known systemic inflammatory disease process (i.e. rheumatoid arthritis), osteoporosis and other metabolic bone diseases, pathological fractures and subsequent non-unions, hypertrophic and infected non-unions. Pregnant women and patients younger than 18 years old and older than 65 years old (for the non-union group) were also excluded from the study.

Dedicated clinics for patients' recruitment and evaluation were set up specifically for this study. The hospital notes and radiographs of all patients recruited were reviewed and such details were documented in a computerized database as patients' demographics, initial fracture pattern, initial treatment received in terms of osteosynthesis, the presence or absence of fracture gap, whether the fracture was closed or open, intake of pharmacological agents, smoking habits, co-morbid conditions and mode of mobilisation. Blood was withdrawn and stored after informed signed consent followed by an interview and a clinical examination.

### DNA isolation

DNA was extracted from peripheral venous blood sample using the QIAamp^® ^DNA Mini Kit (Qiagen, West Sussex, UK). An aliquot of each blood sample was stored at -70°C allowing further DNA extraction, if needed.

### Genes and SNPs selection

Two known BMPs: BMP-2 and BMP-7 and two inhibitory molecules of the BMP pathway: noggin and Smad6 have been selected. BMP-2 gene, located on chromosome 20p12, encompasses 2 exons with a coding region of 1191 nucleotides, produces a protein molecule of 396 amino acids that belongs to the TGF-β superfamily and induces bone and cartilage formation. It has been demonstrated that it is a crucial component for normal fracture healing. Total loss of BMP-2 is lethal; however transgenic mice, in which BMP-2 was inactivated in a limb-specific manner prior to the onset of skeletal development, had spontaneous fractures which did not resolve with time [16]. In particular, it is the earliest steps of fracture healing that seem to be blocked, in the absence of BMP-2, and MSCs at the repair site do not differentiate, leading to a failed healing response. The main role of BMP-2 in fracture healing is highlighted, since its absence could not be compensated efficiently by all the other osteogenic stimuli that were present in the skeleton of these animals [17].

BMP-7 gene (also known as osteogenic protein-1, OP-1), located on human chromosome 20q13, has a coding region of 1296 nucleotides, containing 7 exons and encodes a protein molecule of 431 amino acids that belongs to the TGF-β superfamily and also induces bone and cartilage formation. Its role on the skeleton is suggested from in vivo studies, where BMP-7-deficient mice exhibit skeletal alterations during development. This is restricted to a limited subset of skeletal elements: the rib cage (such as asymmetric pairing of ribs, fusion of ribs, and malformation of the xiphoid process), the skull, and the hind limbs (polydactyly) [18]. On the other hand, BMP-7 null homozygosity in mice is a postnatal lethal condition, associated with various developmental defects, including retarded ossification of bones, fused ribs and vertebrae, and polydactyly [19]. Additionally, in vitro data indicate that BMP-7 possesses different chondrogenic potentials and is more potent than BMP-2 in inducing chondrogenic differentiation of MSCs [20].

NOGGIN gene (NOG), located on human chromosome 17q21-q22, has only one exon of 699 nucleotides, which encodes a protein of 232 amino acids that binds and inactivates BMP signalling. Various animal studies highlight noggin's important role in skeletal physiology. Transgenic mice that over express noggin in osteoblasts exhibit reduced bone mineral densities and bone formation rate, suffer from long bone fractures and osteopenia [21,22]. It has also been shown that exogenous noggin modifies bone formation in adult rats by inhibiting the extent of membranous ossification [23].

SMAD6 gene, located on chromosome 15q21-q22, encompasses 4 exons with a coding region of 1491 nucleotides, producing a protein molecule of 496 amino acids that belongs to the SMAD family of proteins and negatively regulates BMP signalling pathway. Although the SMAD6 in vivo functions are largely unknown, transgenic mice over-expressing SMAD6 showed postnatal dwarfism with osteopenia, impaired bone growth and formation with thin trabecular bone. This is thought to be caused by delayed chondrocyte hypertrophy during endochondral ossification and a reduced population of hypertrophic chondrocytes after birth [24].

Fifteen SNPs of the aforementioned genes of the BMP pathway have been selected to be evaluated. These SNPs had previously been identified and reported in the database of the National Centre for Biotechnology Information http://www.ncbi.nlm.nih.gov/SNP/, with minor allele frequencies greater than 0.2. The SNPs were randomly selected, as there were no previous studies undertaken on this topic to guide selection. For BMP-7, BMP-2 and SMAD6 genes, most SNPs that have been investigated were located in intronic regions. Also, a missense mutation located in exon 3 of BMP-2 was included in the study. Regarding the NOG gene, 3 SNPs were investigated, all of which are located in intragenic regions. Details on the exact position of each SNP within the gene and their nature are summarised in Table 1.

**Table 1:** The selected SNPs (position/function), designed primers and amplicon sizes.

Gene	SNP	SNP position*	Function	Primers Forward/Reverse	Amplicon size
***BMP-2***	rs1005464	intron 2 c.347-2744 G > A	intron	5'-TGAGCGTATATTCCCTAACC-3' 5'-TAACCTCCCAAAAAATTAAATGAC-3'	378 bp
	
	rs235768	exon 3 c.570 A > T	missense mutation (p.R190S)	5'-GCAGAGCTTCAGGTTTTCCG-3' 5'-TGTTTCTCCTCCAAGTGGGC-3'	269 bp
	
	rs235764	intron 2 c.346+3126 G > A	intron	5'-ACTGACATTTTCCGTTCCACCT-3' 5'-TAACAGACAACTGATCAAGGAG-3'	305 bp

***BMP-7***	rs4811822	intron 2 c.612-1290 C > T	intron	5'-CCCAGGGCAACAACAGTCTC-3' 5'-CCTGGGCACACAACTTGACC-3'	260 bp
	
	rs1475000	intron 2 c.611+10288 G > A	intron	5'-TGCAGATGCTGGGTCCTTAA-3' 5'-CGGGTCAGATGCCCATGAAG-3'	281 bp
	
	rs186659	intron 1 c.419-2863 G > A	intron	5'-CTGCAGGGCCTCATACACTA-3' 5'-GAGAACAGCTTCCAGGGTGA-3'	292 bp

***NOG***	rs1442828	intragenic g.13328730 A > G	intragenic	5'-TCCTCTTCGGTCATCCAGTG-3' 5'-TGGTGGAAACCTTGCCATTC-3'	199 bp
	
	rs1372857	intragenic g.13334320 A > G	intragenic	5'-CTGGGAGGGTTCTTGATTGG-3' 5'-ACATGTGAAATGCAGGGCAG-3'	170 bp
	
	rs9915822	intragenic g.13320012 G > T	intragenic	5'-TTAGGCGTCACCCACAGTTG-3' 5'-TGGGCAAGGTAAATGGAAGC-3'	190 bp

***SMAD6***	rs2053423	intron 3 c.13320013 C > T	intron	5'-CATGGCTTGGATGCTTGGTGT-3' 5'-TTCCCAGTCCAAATCAGGGT-3'	398 bp
	
	rs2119261	intron 3 c.952+3144 C > T	intron	5'-GCCACTACTGGACAAACCTT-3' 5'-TCCAACAACTACTCGGCAGA-3'	414 bp
	
	rs3934908	intron 3 c.953-11868 C > T	intron	5'-GAATTGGATGGAGACACGTACC-3' 5'-GATCTGGAATGCTTCCTGAG-3'	542 bp

(* SNP position according to NCBI SNP: Geneview)

### PCR amplification

Primers were designed in close proximity to the selected SNPs and are summarised in Table 1. Amplification of 100 ng of genomic DNA was performed in a 50 μl reaction containing 10 mM Tris HCl pH 9.6, 50 mM KCl, 0.1% v/v Triton-X, 1.5 mM MgCl2, 200 μM dNTPs, 50pmol of each primer and 2.5 units of Taq DNA Polymerase (Promega, Madison, USA). PCR reactions were heated on a PTC-225 Thermal Cycler (MJ Research Inc, USA) at 96°C for 2 minutes, followed by 40 cycles of denaturation at 94°C for 45 seconds, annealing at 55°C for 45 seconds and extension at 72°C for 60 seconds. A final extension step was performed at 72°C for 7 minutes. PCR products were purified using the Exonuclease I/Shrimp Alkaline Phosphatase Method (ExoSAP-IT^®^, USB, Staufen, Germany).

### Sequence Analysis

**Figure 1:**
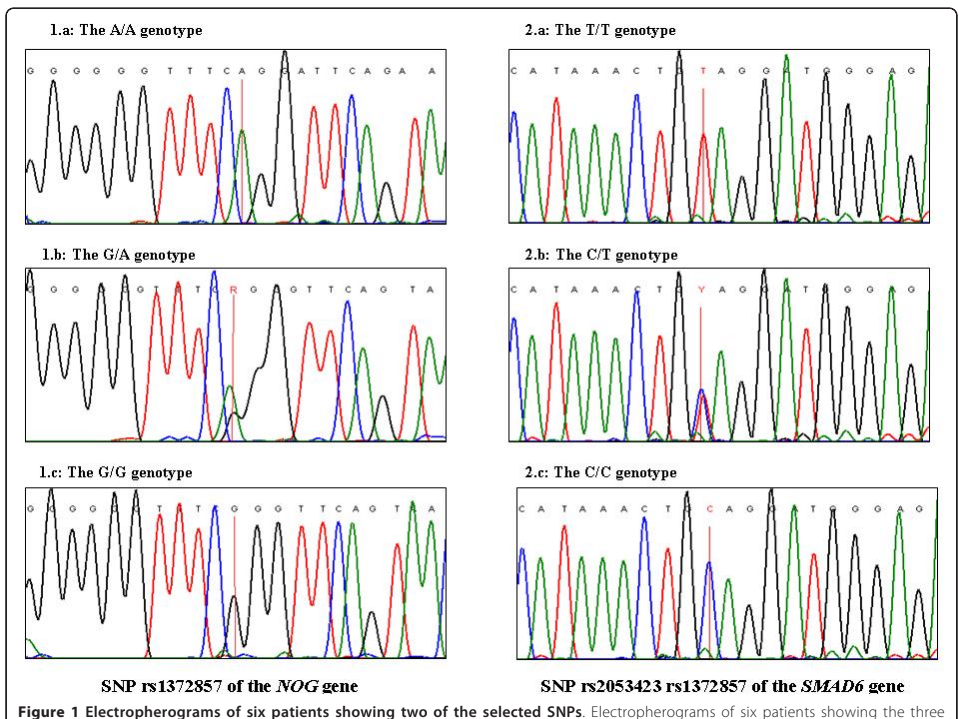
**Electropherograms of six patients showing two of the selected SNPs**. Electropherograms of six patients showing the three possible genotypes of the two SNPs found to be statistically significant: the rs1372857 of the NOG gene (1a, b and c) and the rs2053423 of the SMAD6 gene (2a, b and c).

Automated cycle sequencing for both strands was performed with the BigDye Terminator Cycle Sequencing kit (Applied Biosystems, Warrington, UK). DNA template of 0.2 pmol was mixed with 8 μl sequencing reagent premix and 5 pmol primer and was initially denaturated at 96 °C for 2 minutes, followed by 50 cycles of denaturation at 94 °C for 45 seconds, annealing at 50-55 °C for 45 seconds and extension at 72 °C for 2 minutes. PCR products were then electrophorized in an ABI Prism^® ^3100 Genetic Analyzer (Applied Biosystems, Warrington, UK). Sequences obtained were aligned using the Sequencher^® ^PC software (Gene Codes, USA) with normal sequences taken from Genbank and examined for the presence of polymorphisms (Figure 1).

### Statistical Analysis

Initial exploratory statistical analysis of the various parameters independently, comparing atrophic non-union patients (Group A) with union patients (Group B), was performed using Student's t-test and Pearson's Chi-square test. The overall frequencies of the 15 SNPs were computed for all cases with respect to patient's age, gender, smoking habits, and the use of NSAIDs. Since the outcome measure of interest, namely the non-union or union of the fracture, is dichotomous, a binary logistic regression was used to permit the exploration of many covariates simultaneously [25]. Statistical analysis was performed using STATA 11.1 (StataCorp, Texas USA). A *p *value of 0.05 or less was considered as statistically significant. Odds ratios were calculated in order to evaluate the size of the effect of the tested covariates and describe the strength of their association or non-independence to non-union [26].

## Results

There were sixty-two patients (45 men and 17 women) with atrophic long bone non-unions (Group A/non-union group) with a mean age of 43.9 years (range: 19-65). There were 18 femoral non-unions, 41 tibial, 2 humeral and 1 ulnar non-unions. All these cases required further intervention to achieve union. Control group (Group B/union group) consisted of forty-seven patients (33 men and 14 women) who had uneventful fracture union, with a mean age of 38.4 years (range: 19-78). There was a total of 54 long bone fractures (22 femoral, 26 tibial, 5 humeral and 1 ulnar). The main documented parameters in both groups of patients are summarised in Table 2. The frequencies of all the SNPs genotypes for both groups are summarised in Table 3.

**Table 2:** Individual parameters for patients in Groups A and B and *p *values.

Parameters	Group A (Atrophic non-union)	Group B (Union)	p*	*p^§ ^*[OR, (95% CI)]
Mean age	43.9 yrs	38.4 yrs	**0.025**	**0.01 [1.05, (1.01, 1.08)]**

Sex M/F	45/17	33/14	0.83	0.83 [1.11, (0.44, 2.76)]

Open fractures	45.9%	51.1%	n/a	-

Smoking	46.8%	35.6%	0.32	0.12 [1.99, (0.83, 4.74)]

NSAIDs	38.3%	22.2%	0.09	0.17 [1.92, (0.76, 4.82)]

Fracture comminution	32.2%	27.6%	0.67	-

Segmental fracture	9.7%	2.1%	0.14	-

Bone gap	14.5%	6.4%	0.22	-

Implant failure	6.4%	0	0.13	-

Total number	62	47	n/a	-

[n/a: non applicable, p*: simple statistical analysis using Student's t-test for the parameter of age and Pearson's Chi-square test for the other parameters, ***p^§^***: logistic regression results for individual parameters, odds ratios (OR) and 95% confidence interval (CI)]

**Table 3:** SNPs frequencies and logistic regression results for the non-union status.

Gene	SNP	Genotypes	Group A (non-union)	Group B (union)	Age unadjusted	Age unadjusted	Age adjusted	Age adjusted
					**OR**	***p***	**OR**	***p***

		C/C	20	13	1.00		1.00	
		
	rs4811822	C/T	26	27	0.63	0.30	0.57	0.23
		
		T/T	16	7	1.49	0.49	1.29	0.67
	
		C/C	17	12	1.00		1.00	
		
	rs4811823	C/T	27	27	0.71	0.45	0.61	0.31
		
*BMP-7*		T/T	18	8	1.59	0.42	1.36	0.60
	
		A/A	25	12	1.00		1.00	
		
	rs1475000	G/A	28	28	0.48	0.10	0.48	0.10
		
		G/G	9	7	0.62	0.43	0.70	0.57
	
		A/A	27	15	1.00		1.00	
		
	rs186659	G/A	27	26	0.58	0.19	0.58	0.21
		
		G/G	8	6	0.74	0.63	0.80	0.73
	
		A/A	5	6	1.00		1.00	
		
	rs1005464	G/A	21	11	2.29	0.24	1.90	0.38
		
		G/G	36	30	1.44	0.58	1.13	0.85
	
		A/A	11	9	1.00		1.00	
		
*BMP-2*	rs235768	T/A	31	24	1.06	0.92	1.10	0.86
		
		T/T	20	14	1.17	0.78	1.34	0.62
	
		A/A	8	7	1.00		1.00	
		
	rs235764	G/A	24	13	1.62	0.44	1.67	0.42
		
		G/G	30	27	0.97	0.96	1.21	0.76
	
		A/A	11	4	1.00		1.00	
		
	rs1442828	G/A	33	21	0.57	0.39	0.60	0.45
		
		G/G	18	22	0.30	0.07	0.28	0.06
	
		A/A	16	19	1.00		1.00	
		
*NOG*	rs1372857	G/A	30	24	1.48	0.37	1.63	0.27
		
		G/G	16	4	4.75	**0.02**	4.56	**0.02**
	
		G/G	10	10	1.00		1.00	
		
	rs9915822	G/T	29	27	1.07	0.89	1.28	0.65
		
		T/T	23	10	2.30	0.16	2.26	0.17
	
		C/C	1	5	1.00		1.00	
		
	rs2053423	C/T	25	20	6.25	0.11	7.74	0.09
		
		T/T	36	22	8.18	**0.05**	10.27	**0.04**
	
		C/C	23	20	1.00		1.00	
		
	rs2119261	C/T	34	21	1.41	0.41	1.41	0.41
		
		T/T	5	6	0.72	0.64	1.00	0.99
	
		C/C	36	22	1.00		1.00	
		
*SMAD6*	rs2119260	C/T	25	21	0.73	0.43	0.73	0.45
		
		T/T	1	4	0.15	0.10	0.11	0.07
	
		A/A	2	1	1.00		1.00	
		
	rs3934907	C/A	18	11	0.82	0.88	0.85	0.90
		
		C/C	42	35	0.60	0.68	0.62	0.71
	
		C/C	22	16	1.00		1.00	
		
	rs3934908	C/T	30	24	0.91	0.82	0.95	0.92
		
		T/T	10	7	1.04	0.95	1.05	0.93

[Logistic regression results of genotypes for non-union status: ***p***: *p-*value, **OR: **odds ratios]

Simple statistical analysis using Student's t-test for the parameter of age and Pearson's Chi-square test for the other parameters, including patient's related parameters (gender, smoking, use of NSAIDs), fracture pattern's related factors (comminution, segmental fracture, bone gap) and implant failure, revealed that age was the only statistically significant parameter for the development of fracture non-union with a *p *value of 0.025 (Table 2). In undertaking these separate univariable tests, multiple testing has occurred. As this is only an exploratory study, no adjustment (such as Bonferroni) has been employed.

As the univariate analyses did not allow any adjustment for other factors and in order to assess factors concurrently, a multiple logistic regression of individual parameters in non-union was performed. This yielded adjusted odds ratios (OR) for individual parameters, including age, gender, use of NSAIDs amd smoking (Table 2). Age was found to be an important covariate (*p *= 0.01, OR 1.05 [per year], 95% CI [1.01, 1.08]), whereas the other parameters were found no statistical significant (*p *= 0.83 for gender, *p *= 0.17 for NSAIDs and *p *= 0.12 for smoking). These findings were consistent between unadjusted and adjusted analyses. Therefore, for the consideration of SNPs only age was used as an adjusting covariate, thus simplifying the analysis.

The coefficients from binary logistic regression of the non-union status on the SNP base-pair combinations with and without the inclusion of age as a covariate, based on the previously reported statistical finding are summarised in Table 3. The G/G genotype compared to the A/A genotype of the SNP: rs1372857, located on NOG gene was found to be statistically significant (*p *= 0.02, OR 4.56, age adjusted 95% CI [1.24,16.79]), when union to non-union patients were compared. Specifically, 25.8% from the non-union group were found to be carriers of the G/G genotype compared to the A/A genotype (25.8%), whereas only 8.5% from the union group were found to be carriers of the G/G genotype compared with 40.4% A/A. The same effect (*p *= 0.02, OR 4.75, 95% CI [1.32,17.11]) was observed when analysis was performed without adjusting for age.

The T/T genotype of the SNP: rs2053423 located on SMAD6 gene was noted also to be statistically significant (*p *= 0.04, OR 10.27, age adjusted 95% CI [0.98,107.81]) when compared to the C/C genotype, when union to non-union patients was compared with the age parameter adjusted. Specifically, 58.1% from the non-union group were found to be carriers of the T/T genotype compared to the C/C genotype (1.6%), whereas 46.8% from the union group were found to be carriers of the same genotype compared to 10.7% with the C/C genotype. A similar effect was found to be on the boarders of statistical significance, when analysis was performed without the age parameter adjusted (*p *= 0.05, OR 8.18, 95% CI [0.90,74.69]).

## Discussion

Despite the multi-factorial nature of fracture non-unions, it has been our observation that patients, with comparable fracture patterns and risk factors, may or may not develop non-union. This phenomenon may reflect the presence of a genetic component to impaired bone regeneration and fracture healing. Differences seen in fracture healing response and final outcome therefore may be attributed to biological variations among patients resulting in a 'disturbed' signalling pathway. Genetic variability was found to significantly contribute to the process of bone regeneration [27] and genetic differences between mice strains were shown to affect the length of each stage of fracture healing and the overall healing rate [28]. Therefore, the genetic contribution with or without the interaction of other exogenous factors in cases of impaired fracture healing, is yet to be elucidated. The characterisation of important mediators regulating the fracture healing process, the advances made in diagnostic techniques and the completion of the human genome facilitate the design of studies to explore the potential of disturbed or inhibited physiological processes to be considered in terms of genetic susceptibility. It can therefore be speculated that the "inert or deficient local biology" at the fracture site seen in these cases may represent a genetically predisposed environment with reduced potentials for bone regeneration. Specific genetic variations within the genes involved in fracture healing, and in particular in the BMP signalling pathway therefore, may contribute to the development of atrophic non-union.

In the present study we evaluated a total of fifteen SNPs of specific genes implicated in the BMP pathway, as well as other patient and fracture related factors in the development of atrophic non-unions. Our analysis showed that two specific SNPs and age are statistically significantly associated with atrophic non-union. To the best of our knowledge, this is the first study to investigate the potential of genetic susceptibility to fracture non-union and to suggest a possible genetic association to the impaired bone healing response occurring in these patients. In particular, two genetic polymorphisms in genes involved in the BMP pathway, one found close (~1 Kb) to NOG gene (G/G genotype of the SNP rs1372857) and the other within intron 3 of SMAD6 gene (T/T genotype of the SNP rs2053423) have been associated to the non-union phenotype. Hence, patients with these two particular genotypes may have an increased risk for the development of atrophic non-union. Additionally, an initial exploration of the data revealed that age is an important co-factor and combining the genetic analysis with age as a covariate, a higher impact of our findings was noted.

Noggin, a major antagonist of BMPs, has important functions in respect to bone healing and bone formation. Several mutations of its gene (*NOG*) have been previously reported to be implicated in skeletal anomalies, such as proximal symphalangism, tarsal/carpal coalition syndrome, and brachydactyly type B (BDB) [29,30]. Noggin mutations have also been reported in fibrodysplasia ossificans progressiva (FOP) [31], which is a rare autosomal dominant disorder of skeletal malformations and progressive extraskeletal ossification caused mainly by mutations in the BMP type I receptor ACVR1 [32]. Noggin mutations seem to alter its binding ability to BMPs and growth-differentiation factors (GDFs), thus interfering with the canonical BMP signalling pathway. Animal studies have shown that transgenic mice overexpressing noggin suffered from long bone fractures and osteopenia [21], whereas the knockout noggin mouse was lethal with severe skeletal defects, such as multiple joint fusions [33]. Additionally, exogenous noggin was found to modify bone formation by inhibiting the extent of membranous ossification [23].

The important role of noggin in the BMP cascade is demonstrated by experimental data coming from recent in vitro studies, showing that noggin's reduction promotes osteogenesis by the enhancement of BMP signalling and that addition of noggin successfully blocks in vitro osteogenic differentiation by 50%, resulting in the lowering of BMPs endogenous levels [6,34]. A remarkable finding, highlighting noggin's potential important role in bone healing, was that the bone regenerated closely resembled to normal bone, when the muscle stem cells engineered to express noggin were co-implanted with transduced muscle stem cells producing BMP-4. When the muscle stem cells producing BMP-4 were implanted alone, bone overgrowth was observed. In addition, the co-localisation observed between noggin and BMP-4 during fracture healing, suggests that the balance between them may be an important factor in the regulation of callus formation [35]. Taking into account all reported findings from human, animal and in vitro studies that underline the importance of noggin in skeletal physiology, and in an effort to interpret our results, it can be speculated that specific genetic variations within the NOG gene may be associated with impaired bone healing. In our study, the G/G genotype of the SNP rs1372857 of NOG gene was found to be statistically significant (*p *= 0.02) suggesting a possible association with the defective fracture healing process, seen in atrophic non-unions. A patient with this genotype was found to be of the order of 4 times more likely to develop non-union than a patient with the A/A genotype (OR = 4.75, unadjusted for age 95% CI [1.32,17.11], and OD 4.56, age adjusted 95% CI [1.24,16.79]).

Smad6 is an intracellular inhibitor of the BMP pathway. Although its in vivo functions are largely unknown, transgenic mice overexpressing Smad6 in chondrocytes showed postnatal dwarfism with osteopenia and impaired bone growth and formation, caused by delayed chondrocyte hypertrophy during endochondral ossification [24]. These findings may indicate a distinct role for Smad6 in bone regeneration. However, there are no human genetic data correlating SMAD6 mutations with syndromes with skeletal involvement, except from a recently published study assessing the role of the SMAD6 in the regulation of bone mass. Association analysis between bone mineral density and SMAD6 SNPs in 721 Japanese postmenopausal women identified a specific SNP (rs755451), located on intron 3 of SMAD6, to be associated with lower bone mineral density in postmenopausal women, and thus increasing the risk of osteoporosis. This finding marks the regulatory role of SMAD6 in bone homeostasis [36]. In the present study, logistic regression showed that the T/T genotype of the rs2053423 SNP within the same intron (intron 3) of SMAD6 was more frequent compared to the C/C genotype within non-union patients, with an odds ratio of ≈ 8 and 10 (after age adjustment) for fracture patients to develop non-union if they have the T/T genotype of this particular SNP compared to the C/C genotype.

In the herein study, statistical analysis of other factors known to predispose to atrophic non-union did not reveal any association except the covariate of age, with an odds ratio of 1.05 for one year of age. This can be attributed to the effect of aging at the cellular level. Although, MSCs were reported to maintain their differentiation potential during aging [37], researchers found aging to be associated with decreased proliferative capacity of osteoprogenitor cells and therefore with a decreased osteoblastic cell number and osteoblastogenesis [38].

### Study limitations

The sample size of patients that were included is relatively small to perform a fully powered statistical analysis and to make firm conclusions about the likelihood of genetic predisposition to fracture non-union. Also, the heterogeneity of the two groups regarding the mechanism and pattern of the fractures, the use of NSAIDs, smoking, the different treatment options, as well as the small number of SNPs and genes involved in fracture healing that were evaluated, could be considered as additional limitations of this case control association study. Thus, it may be possible that some associations have not been detected and the role of other genes has not been evaluated. In addition, due to the high cost of genetic profiling, it was not possible to perform a full cohort study or to examine a larger number of SNPs either for the selected genes or for a number of other genes known to be expressed during fracture healing. However, it should be noted that this study was undertaken as a pilot study based on observational data, aiming to explore a potential impact of genetic variations on the development of fracture non-union and to stimulate further research into a number of candidate genes of the bone healing cascade. A second limitation of the present study is that tag SNPs have not been considered and most SNPs that have been investigated were located in intronic or intragenic regions. This selection was made, because the majority of the published exonic sequence variants for the selected genes were either synonymous or frame shift or nonsense mutations, suggesting that they were pathogenic mutations. However, these intronic or intragenic SNPs, especially for the relatively small genes that have been investigated in this study, may be linked with genetic variants within the coding and regulatory regions of these genes due to the phenomenon of linkage disequilibrium. In general, the actual association between genes and disease, and especially in polygenic and exogenously affected traits, can be performed using intronic or intragenic variants, commonly observed within the general population, as tags for the identification of the actual predisposing variants [39,40]. In general, the BMP signalling pathway is implicated in a variety of processes during development and adult life, and is expressed in different tissues besides bone. Therefore, no major genetic alterations, if any, within the BMP signalling genes are expected to be found, even if genetic predisposition to atrophic non-union does exist. Important mutations of these genes are linked to major phenotype alterations and defects, and even lethal conditions. However, multiple low penetrance alleles (each with a small effect) may interact and be associated with the defective bone regeneration, seen in atrophic non-unions. Furthermore, since no previous work has been published assessing the genetic predisposition for the development of atrophic non-union (using SNPs or any other genetic markers), this pilot study aimed to explore a possible genetic impact by selecting a small initial number of SNPs located on specific genes implicated in fracture healing.

With the herein preliminary study, the analysis can only be exploratory, aiming to suggest candidate covariates for further studies and stimulate future research. Larger trials may determine that a number of genetic variants in combination with other known factors may be influential in the healing of fractures and more subtle effects may be revealed. The genetic input to the impaired fracture healing may be determined by the genotyping of exonic polymorphisms not only within the already short-listed genes, but also within other genes involved in the complex cascade of bone healing. Advanced technology, such as custom-built microarrays can be very helpful and can simultaneously examine a large number of SNPs within the numerous genes expressed during fracture healing, in order to identify functional polymorphisms as well as influential combinations of SNPs with a higher predictive power.

### Clinical relevance

From the clinical perspective, analysis of SNPs linked to aberrant bone healing can be used as a potential powerful tool to rapidly identify patients at risk of developing atrophic fracture non-union. As most fractures unite uneventfully, one may argue that it may not be cost effective to subject all fracture patients to genetic testing. However, such analysis may be valuable in patients that demonstrate slow progression to union or no progression at all, especially in the absence of other known risk factors or in particular in the older patient with a long bone fracture non-union. In these cases, early intervention to augment the local biology for bone regeneration, could facilitate the union of the fracture and even accelerate the time to union. Consequently, greater knowledge of the genes involved in fracture repair may provide new approaches at the molecular level in the treatment of these patients and the on-time intervention in the biologic aspects of bone healing. Currently, biological response modifiers are already in clinical use or under extensive investigation as alternatives or adjuvants for the management of defective bone healing. Specifically, with the use of recombinant technology, BMP-2 and BMP-7 are available for implantation for acceleration or stimulation of bone regeneration in cases of open fractures and atrophic fracture non-unions, respectively [41,42]. However, these currently available treatment modalities do not address the issue of possible isolated gene disturbances. There are novel methods such as gene therapy (with local or systemic administration) and tissue engineering, which aim to address such issues, but are still under investigation. If genetic predisposition to atrophic non-union does exist, such expensive modalities may be used to selected (after genetic testing) patients.

## Conclusion

This pilot study investigated the possible impact of genetic predisposition to atrophic fracture non-union, by assessing a number of candidate genes of the different signalling pathways of fracture healing, and suggests the potential existence of a genetically predetermined impairment within the BMP signaling cascade, initiated after a fracture and when combined with other risk factors could synergistically increase the susceptibility of a patient to develop non-union. The genetic component and its role and interaction with other risk factors in the development of atrophic fracture non-unions merit further investigation.

## Competing interests

The authors declare that they have no competing interests.

## Authors' contributions

RD is the main author of this paper, carried out the laboratory work (DNA extraction, PCR, sequencing) as well as the recruitment and clinical and radiological assessment of the patients and the analysis and interpretation of the data. IMC supervised and also contributed in the laboratory work, participated in the design of the study and helped to draft the manuscript. His contribution was also in the analysis and interpretation of the results. RBW made substantial contributions to the analysis, interpretation and presentation of data; by performing the statistical analysis and helping to write the results of the study. AFM made substantial contributions to conception and design of the study, and participated in its coordination. PVG made substantial contributions to conception and design of the study, acquisition and interpretation of data, and coordinated the study. He helped to draft the manuscript and revised it critically for important intellectual content. All authors read and have given final approval of the final manuscript.

## Pre-publication history

The pre-publication history for this paper can be accessed here:

http://www.biomedcentral.com/1471-2474/12/44/prepub

## References

[B1] Praemer A Furner S Rice DP From Musculoskeletal injuries American Academy of Orthopaedic Surgeons. Musculoskeletal conditions in the United States 1992 Park Ridge, IL, USA 85 124

[B2] Brinker MR Browner BD, Jupiter JB, Levine AM, Trafton PG From Nonunions: evaluation and treatment Skeletal Trauma Basic science management and reconstruction 2003 3 Philadelphia: Saunders 507 604

[B3] Giannoudis PV MacDonald DA Matthews SJ Smith RM Furlong AJ De Boer P Nonunion of the femoral diaphysis. The influence of reaming and non-steroidal anti-inflammatory drugs J Bone Joint Surg Br 2000 82 5 655 658 10.1302/0301-620X.82B5.9899 10963160

[B4] Cuthbert AP Fisher SA Mirza MM King K Hampe J Croucher PJ Mascheretti S Sanderson J Forbes A Mansfield J Schreiber S Lewis CM Mathew CG The contribution of NOD2 gene mutations to the risk and site of disease in inflammatory bowel disease Gastroenterology 2002 122 4 867 874 10.1053/gast.2002.32415 11910337

[B5] Hyndman ME Parsons HG Verma S Bridge PJ Edworthy S Jones C Lonn E Charbonneau F Anderson TJ The T-786-- > C mutation in endothelial nitric oxide synthase is associated with hypertension Hypertension 2002 39 4 919 922 10.1161/01.HYP.0000013703.07316.7F 11967250

[B6] Raunio H Husgafvel-Pursiainen K Anttila S Hietanen E Hirvonen A Pelkonen O Diagnosis of polymorphisms in carcinogen-activating and inactivating enzymes and cancer susceptibility--a review Gene 1995 159 1 113 121 10.1016/0378-1119(94)00448-2 7607565

[B7] Ralston SH Genetics of osteoporosis Proc Nutr Soc 2007 66 2 158 165 10.1017/S002966510700540X 17466098

[B8] Akbarian S Huang HS Molecular and cellular mechanisms of altered GAD1/GAD67 expression in schizophrenia and related disorders Brain Res Rev 2006 52 2 293 304 10.1016/j.brainresrev.2006.04.001 16759710

[B9] Nakamura Y DNA variations in human and medical genetics: 25 years of my experience J Hum Genet 2009 54 1 1 8 10.1038/jhg.2008.6 19158818

[B10] Dimitriou R Tsiridis E Giannoudis PV Current concepts of molecular aspects of bone healing Injury 2005 36 12 1392 1404 10.1016/j.injury.2005.07.019 16102764

[B11] Gazzerro E Canalis E Bone morphogenetic proteins and their antagonists Rev Endocr Metab Disord 2006 7 1-2 51 65 10.1007/s11154-006-9000-6 17029022

[B12] Yamashita H Ten Dijke P Heldin CH Miyazono K Bone morphogenetic protein receptors Bone 1996 19 569 574 10.1016/S8756-3282(96)00259-1 8968021

[B13] Miyazono K Signal transduction by bone morphogenetic protein receptors: functional roles of Smad proteins Bone 1999 25 91 93 10.1016/S8756-3282(99)00113-1 10423029

[B14] Dimitriou R Tsiridis E Carr I Simpson H Giannoudis PV The role of inhibitory molecules in fracture healing Injury 2006 37 Suppl 1 20 29 10.1016/j.injury.2006.02.039 16616754

[B15] Abe E Yamamoto M Taguchi Y Lecka-Czernik B O'Brien CA Economides AN Stahl N Jilka RL Manolagas SC Essential requirement of BMPs-2/4 for both osteoblast and osteoclast formation in murine bone marrow cultures from adult mice: antagonism by noggin J Bone Miner Res 2000 15 4 663 673 10.1359/jbmr.2000.15.4.663 10780858

[B16] Zhang H Bradley A Mice deficient for BMP2 are nonviable and have defects in amnion/chorion and cardiac development Development 1996 122 2977 2986 8898212 10.1242/dev.122.10.2977

[B17] Tsuji K Bandyopadhyay A Harfe BD Cox K Kakar S Gerstenfeld L Einhorn T Tabin CJ Rosen V BMP2 activity, although dispensable for bone formation, is required for the initiation of fracture healing Nat Genet 2006 38 12 1424 1429 10.1038/ng1916 17099713

[B18] Luo G Hofmann C Bronckers AL Sohocki M Bradley A Karsenty G BMP-7 is an inducer of nephrogenesis, and is also required for eye development and skeletal patterning Genes Dev 1995 9 22 2808 2820 10.1101/gad.9.22.2808 7590255

[B19] Jena N Martín-Seisdedos C McCue P Croce CM BMP7 null mutation in mice: developmental defects in skeleton, kidney, and eye Exp Cell Res 1997 230 1 28 37 10.1006/excr.1996.3411 9013703

[B20] Shintani N Hunziker EB Chondrogenic differentiation of bovine synovium: bone morphogenetic proteins 2 and 7 and transforming growth factor beta1 induce the formation of different types of cartilaginous tissue Arthritis Rheum 2007 56 6 1869 1879 10.1002/art.22701 17530715

[B21] Devlin RD Du Z Pereira RC Kimble RB Economides AN Jorgetti V Canalis E Skeletal overexpression of noggin results in osteopenia and reduced Bone formation Endocrinology 2003 144 5 1972 1978 10.1210/en.2002-220918 12697704

[B22] Wu XB Li Y Schneider A Yu W Rajendren G Iqbal J Yamamoto M Alam M Brunet LJ Blair HC Zaidi M Abe E Impaired osteoblastic differentiation, reduced bone formation, and severe osteoporosis in noggin-overexpressing mice J Clin Invest 2003 112 6 924 934 12975477 10.1172/JCI15543 PMC193662

[B23] Aspenberg P Jeppsson C Economides AN The Bone morphogenetic proteins antagonist noggin inhibits membranous ossification J Bone Miner Res 2001 16 3 497 500 10.1359/jbmr.2001.16.3.497 11277267

[B24] Horiki M Imamura T Okamoto M Hayashi M Murai J Myoui A Ochi T Miyazono K Yoshikawa H Tsumaki N Smad6/Smurf1 overexpression in cartilage delays chondrocytes hypertrophy and causes dwarfism with osteopenia J Cell Biol 2004 165 433 445 10.1083/jcb.200311015 15123739 PMC2172180

[B25] Setakis E Stirnadel H Balding DJ Logistic regression protects against population structure in genetic association studies Genome Res 2006 169 2 290 226 10.1101/gr.4346306 PMC1361725 16354752

[B26] Bland JM Altman DG Statistics notes. The odds ratio BMJ 2000 320 7247 1468 10.1136/bmj.320.7247.1468 10827061 PMC1127651

[B27] Manigrasso MB O'Connor JP Comparison of fracture healing among different inbred mouse strains Calcif Tissue Int 2008 82 6 465 474 10.1007/s00223-008-9144-3 18528610

[B28] Jepsen KJ Hu B Tommasini SM Courtland HW Price C Terranova CJ Nadeau JH Genetic randomization reveals functional relationships among morphologic and tissue-quality traits that contribute to bone strength and fragility Mamm Genome 2007 18 6-7 492 507 10.1007/s00335-007-9017-5 17557179 PMC1998883

[B29] Dixon ME Armstrong P Stevens DB Bamshad M Identical mutations in NOG can cause either tarsal/carpal coalition syndrome or proximal symphalangism Genet Med 2001 3 5 349 353 10.1097/00125817-200109000-00004 11545688

[B30] Lehmann K Seemann P Silan F Goecke TO Irgang S Kjaer KW Kjaergaard S Mahoney MJ Morlot S Reissner C Kerr B Wilkie AO Mundlos S A new subtype of brachydactyly type B caused by point mutations in the bone morphogenetic protein antagonist NOGGIN Am J Hum Genet 2007 81 2 388 396 10.1086/519697 17668388 PMC1950796

[B31] Sémonin O Fontaine K Daviaud C Ayuso C Lucotte G Identification of three novel mutations of the noggin gene in patients with fibrodysplasia ossificans progressiva Am J Med Genet 2001 102 4 314 317 11503156 10.1002/ajmg.1504

[B32] Shore EM Xu M Feldman GJ Fenstermacher DA Cho TJ Choi IH Connor JM Delai P Glaser DL LeMerrer M Morhart R Rogers JG Smith R Triffitt JT Urtizberea JA Zasloff M Brown MA Kaplan FS A recurrent mutation in the BMP type I receptor ACVR1 causes inherited and sporadic fibrodysplasia ossificans progressive Nat Genet 2006 38 5 525 527 10.1038/ng1783 16642017

[B33] Brunet LJ McMahon JA McMahon AP Harland RM Noggin, cartilage morphogenesis, and joint formation in the mammalian skeleton Science 1998 280 5368 1455 1457 10.1126/science.280.5368.1455 9603738

[B34] Edgar CM Chakravarthy V Barnes G Kakar S Gerstenfeld LC Einhorn TA Autogenous regulation of a network of bone morphogenetic proteins (BMPs) mediates the osteogenic differentiation in murine marrow stromal cells Bone 2007 40 5 1389 1398 10.1016/j.bone.2007.01.001 17303481 PMC2681090

[B35] Yoshimura Y Nomura S Kawasaki S Tsutsumimoto T Shimizu T Takaoka K Colocalization of noggin and bone morphogenetic protein-4 during fracture healing J Bone Miner Res 2001 16 5 876 884 10.1359/jbmr.2001.16.5.876 11341332

[B36] Urano T Shiraki M Usui T Sasaki N Ouchi Y Inoue S Bone mass effects of a Smad6 gene polymorphism in Japanese postmenopausal women J Bone Miner Metab 2009 27 5 562 566 10.1007/s00774-009-0068-4 19277452

[B37] Justesen J Stenderup K Eriksen EF Kassem M Maintenance of osteoblastic and adipocytic differentiation potential with age and osteoporosis in human marrow stromal cell cultures Calcif Tissue Int 2001 71 1 36 44 10.1007/s00223-001-2059-x 12200657

[B38] Jiang Y Mishima H Sakai S Liu YK Ohyabu Y Uemura T Gene expression analysis of major lineage-defining factors in human bone marrow cells: Effect of aging, gender, and age-related disorders J Orthop Res 2008 26 7 910 917 10.1002/jor.20623 18302252

[B39] Garcia-Closas M Troester MA Qi Y Langerød A Yeager M Lissowska J Brinton L Welch R Peplonska B Gerhard DS Gram IT Kristensen V Børresen-Dale AL Chanock S Perou CM Common genetic variation in GATA-binding protein 3 and differential susceptibility to breast cancer by estrogen receptor alpha tumor status Cancer Epidemiol Biomarkers Prev 2007 16 11 2269 2275 10.1158/1055-9965.EPI-07-0449 18006915

[B40] Vonlanthen S Kawecki TJ Betticher DC Pfefferli M Schwaller B Heterozygosity of SNP513 in intron 9 of the human calretinin gene (CALB2) is a risk factor for colon cancer Anticancer Res 2007 27 6C 4279 4288 18214032

[B41] Govender S Csimma C Genant HK Valentin-Opran A Amit Y Arbel R Aro H Atar D Bishay M Börner MG Chiron P Choong P Cinats J Courtenay B Feibel R Geulette B Gravel C Haas N Raschke M Hammacher E van der Velde D Hardy P Holt M Josten C Ketterl RL Lindeque B Lob G Mathevon H McCoy G Marsh D Miller R Munting E Oevre S Nordsletten L Patel A Pohl A Rennie W Reynders P Rommens PM Rondia J Rossouw WC Daneel PJ Ruff S Rüter A Santavirta S Schildhauer TA Gekle C Schnettler R Segal D Seiler H Snowdowne RB Stapert J Taglang G Verdonk R Vogels L Weckbach A Wentzensen A Wisniewski T BMP-2 Evaluation in Surgery for Tibial Trauma (BESTT) Study Group Recombinant human bone morphogenetic protein-2 for treatment of open tibial fractures: a prospective, controlled, randomized study of four hundred and fifty patients J Bone Joint Surg Am 2002 84 12 2123 2134 12473698 10.2106/00004623-200212000-00001

[B42] Friedlaender GE Perry CR Cole JD Cook SD Cierny G Muschler GF Zych GA Calhoun JH LaForte AJ Yin S Osteogenic protein-1 (bone morphogenetic protein-7) in the treatment of tibial nonunions J Bone Joint Surg Am 2001 83 Suppl A 151 158 11314793 PMC1425155

